# Biomarkers for rhythmic and discrete dynamic primitives in locomotion

**DOI:** 10.1038/s41598-022-24565-z

**Published:** 2022-11-23

**Authors:** Rui Moura Coelho, Hiroaki Hirai, Jorge Martins, Hermano Igo Krebs

**Affiliations:** 1 IDMEC, Instituto Superior Técnico, Universidade de Lisboa, Av. Rovisco Pais 1, 1049-001 Lisbon, Portugal; 2 Department of Mechanical Science and Bioengineering, Osaka University, Suita, 565-0871 Japan; 3 Department of Mechanical Engineering, Massachusetts Institute of Technology, Cambridge, 02139 USA

**Keywords:** Biomedical engineering, Diagnostic markers, Diagnosis, Rehabilitation, Disability

## Abstract

Rehabilitation can promote brain plasticity and improve motor control after central nervous system injuries. Our working model is that motor control is encoded using dynamic primitives: submovements, oscillations, and mechanical impedances. We hypothesize that therapies focusing on these primitives can achieve greater motor recovery. At the observational level, these primitives lead to discrete and rhythmic movements. Here, we propose two novel biomarkers to evaluate rhythmic and discrete movements in gait based on the feet forward position: the smoothness of their relative position, using the mean-squared jerk ratio (MSJR), to assess rhythmicity; and the angle between principal components of consecutive trajectories (dPCA), to detect discrete movements amidst rhythmic motion. We applied these methods to kinematic data collected with healthy individuals during experiments employing the MIT-Skywalker: level-ground walking at five speeds, with and without imposed ankle stiffness; walking at constant speed on ascending, descending, and laterally tilted slopes; and performing sidesteps. We found a decrease in MSJR as speed increases, related to increased rhythmicity, even with imposed stiffness. Rhythmicity seems unaffected by the terrain perturbations imposed. Finally, dPCA successfully detects sidesteps, discrete events amidst rhythmic movement. These biomarkers appear to accurately assess rhythmic and discrete movements during walking and can potentially improve clinical evaluation and rehabilitation of neurological patients.

## Introduction

Injuries to the central nervous system often have deleterious effects on motor function. Stroke is the most common of these injuries resulting in almost 800,000 new injuries per year in the USA alone, of which $80\%$ will have some form of motor impairment^1^. Depending on the size and location of the lesion, movement impairment can occur in the upper limb, lower limb, or both. There are several mechanisms of post-stroke recovery. Initially during the acute phase, there is the resolution of harmful local factors and edema. The recovery of partially damaged nerves and clearance of cytotoxins can result in large motor improvements^2^. The second mechanism of recovery involves neuroplasticity, the ability of the nervous system to modify its structural and functional organization either through sprouting of collateral synaptic connections to de-innervated tissue or the usage of previously latent neural pathways^3^. Multiple studies have shown that in both humans and animals this restructuring is use-dependent and benefits from both forced use and functional training, and it might occur during the acute, sub-acute, and chronic stroke^3^. The neural plasticity benefits resulting from experience dependent training is part of the reason justifying the attempts to reduce impairments rather than using exclusively techniques that promote substitution and compensation^3^. While many different methods of physical therapy exist, the employment of robotics during training is a rapidly developing field. For the upper extremity, robot-assisted therapy is recommended since 2010 as an adjunct treatment by the American Heart Association^4,5^. One such device, the MIT-Manus, has been shown to provide long-lasting benefits to motor function when used in both acute and chronic phases of stroke as compared with usual care^6–14^. Currently, the same cannot be said of the lower extremity (LE); both the American Heart Association and the Veterans Administration guidelines for post-stroke care do not endorse the use of robotic devices since they have yet to demonstrate any advantage over usual care as practiced in the US^4,15^. This apparent immaturity of LE robotic therapy reflects the fact that, to date, knowledge of human motor control has not been applied to LE robotic therapy. Our final goal is to codify a competent model that is customizable to individuals with different biomechanical and impairment characteristics. We envision the customized models providing a rationale for targeting LE therapy and assistive devices to specific deficits identified within the model framework, hence improving outcomes. The MIT-Skywalker is an attempt to fill this gap in post-stroke treatment affording therapeutic training focused on purported primitives of movement, namely: oscillations, submovements, and mechanical impedance^16–20^.

These dynamics primitives of motion represent our working model on how the central nervous system (CNS) commands and controls human movement^21,22^. These primitives lead, at the end-effector observational level, to discrete and rhythmic movements^17^.

Discrete movements are, by definition, movements of non-negligible duration, preceded and succeeded by distinct postures, i.e., periods of no movement. Rhythmic movements can be described as an attractor that exhibits almost periodic motion. To generate motion, we consider the conjecture that the CNS is commanding a virtual trajectory for the limbs to follow, composed of a combination of submovements and/or oscillations^18,20^. A mechanical impedance primitive drives the limb towards the virtual trajectory and might also account for stable contact with the environment (for more details of our working model see Supplementary Fig. S1). There has been a great effort to develop approaches to identify the ankle mechanical impedance in healthy and stroke patients^19,23–27^. Here, we will examine approaches to differentiate discrete from rhythmic movements.

Evidence suggests that a rhythmic movement is not a composition of discrete movements but it is a primitive on its own, as they recruit different areas of the brain^28^, and thus injuries may affect these primitives differently^29^. Therapy may be designed to promote the improvement of a particular deficit associated with a particular primitive. The concept of rhythmicity and discreteness of movements has been extensively studied for the upper limb^20,30–33^, with several metrics being proposed to characterize them. Smoothness of movement in both end-effector and joint coordinates can be used to quantify rhythmicity^20,29,31,34^. Previous approaches characterize gait rhythmicity based on temporal features of gait, such as stride and swing time variability, gait asymmetry or phase coordination^35–41^. Such temporal features do not account for the kinematics of movement, which is of particular importance in the scope of our dynamic primitives model. This work introduces a novel methodology to assess the rhythmicity and discreteness of walking gait using kinematic data, suitable for different walking scenarios, and to detect discrete events that might occur amidst rhythmic gait. The MIT-Skywalker was used in this study to allow for a range of different walking scenarios^16^. We hypothesize that walking gait on level ground at low speeds is a sequence of discrete movements and that, as speed increases, the movement becomes more rhythmic. Furthermore, the introduction of slopes or laterally tilted surfaces should not affect rhythmicity when walking at a comfortable speed, but introduce some discrete components. In addition, we assume that a sidestep occurring amidst a rhythmic walking gait is a discrete event. Finally, considering our working model, where the virtual trajectory is composed of a combination of submovements and oscillations, and the mechanical impedance is an independent primitive that attracts the foot towards this trajectory, we expect that an externally imposed ankle stiffness will not affect the virtual trajectory of the foot, and hence rhythmicity or discreteness of gait should not be affected.

Walking gait is mostly periodic at any given speed, although at slower speeds it will present periods of posture, where both feet are immobile on the ground^41^. As speed increases, this period will be greatly reduced, and the relative position between the two feet along time will become closer to a sinusoidal wave, which would represent an ideal rhythmic behavior. We propose taking the smoothness of the relative position between the two feet to analyze rhythmicity, computing the mean-squared jerk ratio (MSRJ) between the movement and an ideally rhythmic behavior. The MSJR can be associated to a level of rhythmicity or discreteness of the resulting movement^34^.

Evaluating the forward position of one foot against another in a 2D graph, a figure-8 pattern arises, highly repeatable in unimpaired subjects, but also in patients with spinal cord injuries during body-weight support (but not unloading) treadmill training^42,43^. This 2D rhythmic spatial trajectory encodes interlimb coordination, unlike other cyclic representations such as cyclograms^44^. A discrete movement, such as a sidestep, will disrupt the typical pattern and hence it is detectable by an increase in the angle between the principal components of the trajectories in consecutive steps (dPCA).

## Results

The algorithms to compute mean-squared jerk ratio, MSJR, and the angle difference between the Principal Components of the spatial trajectories, dPCA, described in “Methods”, were applied to the data from the different experiments. The kinematic data included in this study is summarized in Supplementary Table S1, which presents the number of strides considered for each subject in each experiment.

### MSJR

**Figure 1:**
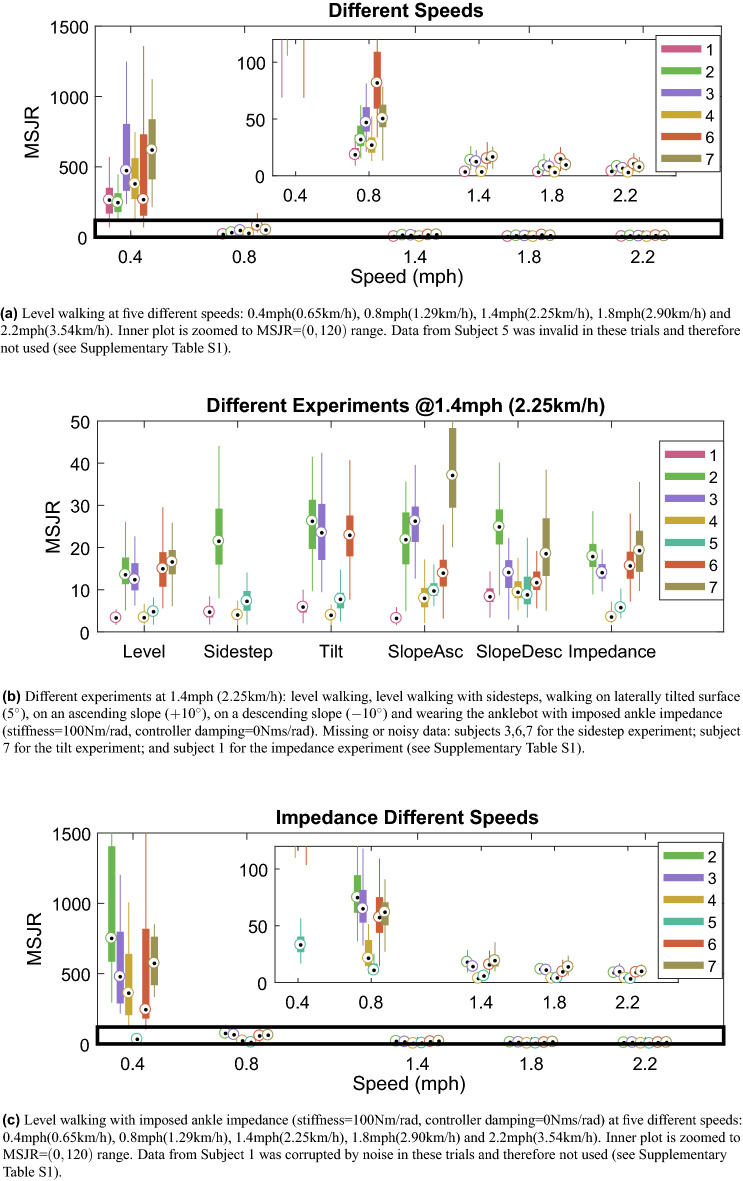
Box-plots of the mean-squared jerk ratio (MSJR) for each subject.

**Figure 2:**
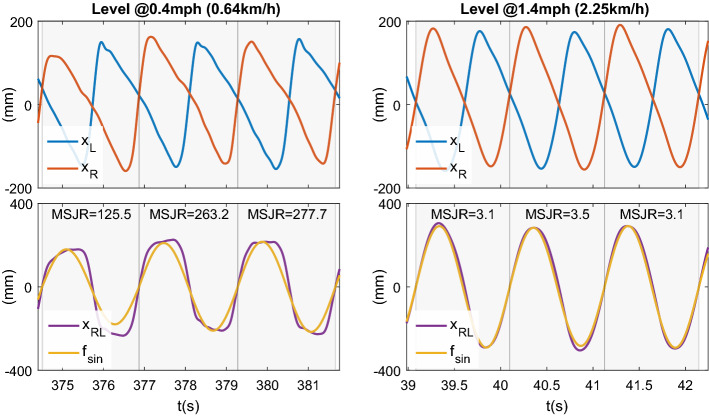
Example of left ($x_L$) and right ($x_R$) foot position time evolution for two speeds, in level ground walking. The relative position between both feet, $x_{RL}$ is taken to compute the MSJR with respect to a sine wave, $f_{sin}$, a maximally smooth rhythmic movement.

The box-plots for the computed MSJR for the different trials are shown in Fig. 1. Figure 2 shows the kinematic data from three strides of a subject walking on level ground at 0.4 mph (0.64 km/h) and at 1.4 mph (2.25 km/h), and the MSJR for each stride. Level walking at different speeds (Fig. 1a) reveals a common trend for all subjects: high MSJR ($>150$) at the lowest speed and monotonically decreasing as the speed increases. For speeds $\ge 1.4$ mph ($\sim 2.25$ km/h), MSJR remains constant or slightly decreases below 20 for every subject. At 0.8 mph ($\sim 1.29$ km/h), the median MSJR values assumed are highly dependent on the subject, ranging from 20 to 100. For level-ground walking with imposed impedance at different speeds (Fig. 1c), MSJR values are similar to those found in free walking. At 0.8 mph (1.29 km/h), MSJR increases slightly for each subject when compared to free walking trials, although still within the same range of values. The two-way repeated measures analysis of variance (ANOVA) for MSJR revealed a statistically significant effect of Speed (F$_{1.015, 4.060}=41.073$, $p=0.003$), but not of ankle Impedance (F$_{1, 4}=0.857$, $p=0.407$). Furthermore, the interaction effect between Speed and ankle Impedance was not significant (F$_{1.007, 4.029}=0.753$, $p=0.435$). Analyzing the effect of Speed, there is no statistically significant difference between speeds 1.4, 1.8 and 2.2 mph (2.25, 2.90, 3.54 km/h) $(p>0.22)$, with MSJR close to 9.95, and significant differences otherwise. The mean difference in MSJR between 0.4 mph (0.65 km/h) and 1.4, 1.8 and 2.2 mph (2.25, 2.90, 3.54 km/h) is 409.61, and between 0.8 mph (1.29 km/h) and 1.4, 1.8 and 2.2 mph (2.25, 2.90, 3.54 km/h) is 41.15. Finally, there is a mean difference in MSJR of 368.46 between speeds 0.4 and 0.8 mph (0.65 and 1.29 km/h).

**Figure 3:**
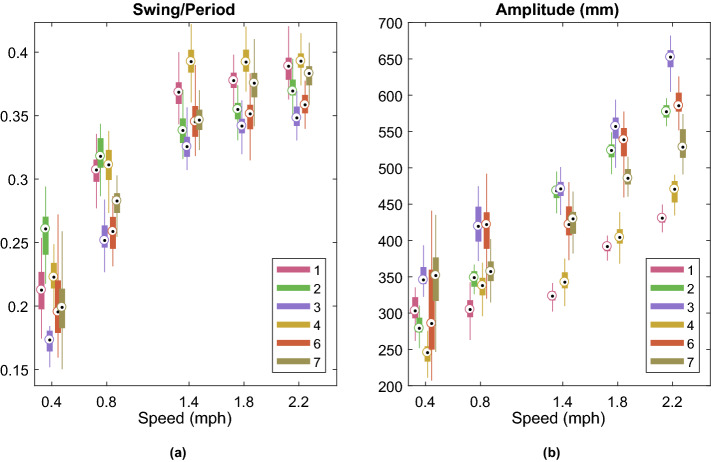
Features from level-ground walking at different speeds, 0.4 mph (0.65 km/h), 0.8 mph (1.29 km/h), 1.4 mph (2.25 km/h), 1.8 mph (2.90 km/h) and 2.2 mph (3.54 km/h), for each subject: (**a**) stride swing/period ratio; (**b**) step amplitude. Data from Subject 5 was invalid in these trials and therefore not used (see Supplementary Table S1).

In order to explain the range of MSJR values found at the different speeds, the step amplitude and the ratio between swing time and stride period were computed for all strides of every subject in level walking at different speeds (Fig. 3). Swing time is computed from the forward position as the time between a negative peak and the following positive peak. The difference in forward position between these two points is the step amplitude. The period is computed as described in “Methods” for the sine wave period. The stride swing/period ratio (Fig. 3a) follows the same trend for each subject, increasing monotonically with speed, approaching 0.4 as the speed increases, with inter-subject variability. For speeds $\ge 1.4$ mph ($\sim 2.25$ km/h), the ratio tends to stabilize or increase slightly for every subject. The step amplitude (Fig. 3b) tends to increase with speed, although between 0.4 and 0.8 mph (0.65 and 1.29 km/h) and between 0.8 and 1.4 mph (1.29 and 2.25 km/h), some subjects keep a constant amplitude. However, from 1.4 mph (2.25 km/h) onwards, the amplitude increase seems to follow a linear model in every subject, unlike the stride swing/period ratio.

The MSJR computed for the different experiments performed at 1.4 mph (2.25 km/h) is presented in Fig. 1b, showing that the median MSJR remains below 30 for all experiments, hence within the range of values for level-walking. The ANOVA revealed no significant difference between experiments (F$_{2.712, 10.849}=3.225$, $p=0.069$). Harmonicity was computed for level-ground walking at different speeds (Supplementary Fig. S2)^33^. Although harmonicity is low ($<0.3$) for speeds $\le 0.8$ mph ($\sim 1.29$ km/h), its median value is low as speed increases, and its variability high, lying within the complete range of possible values (between 0 and 1).

### dPCA

A typical 2D spatial trajectory of the forward position of both feet resulting from many strides in level ground walking is shown in Supplementary Fig. S3 for five different speeds. The amplitude of the orbits increases with speed, but the main direction of the principal components remains relatively constant across speeds.

**Figure 4:**
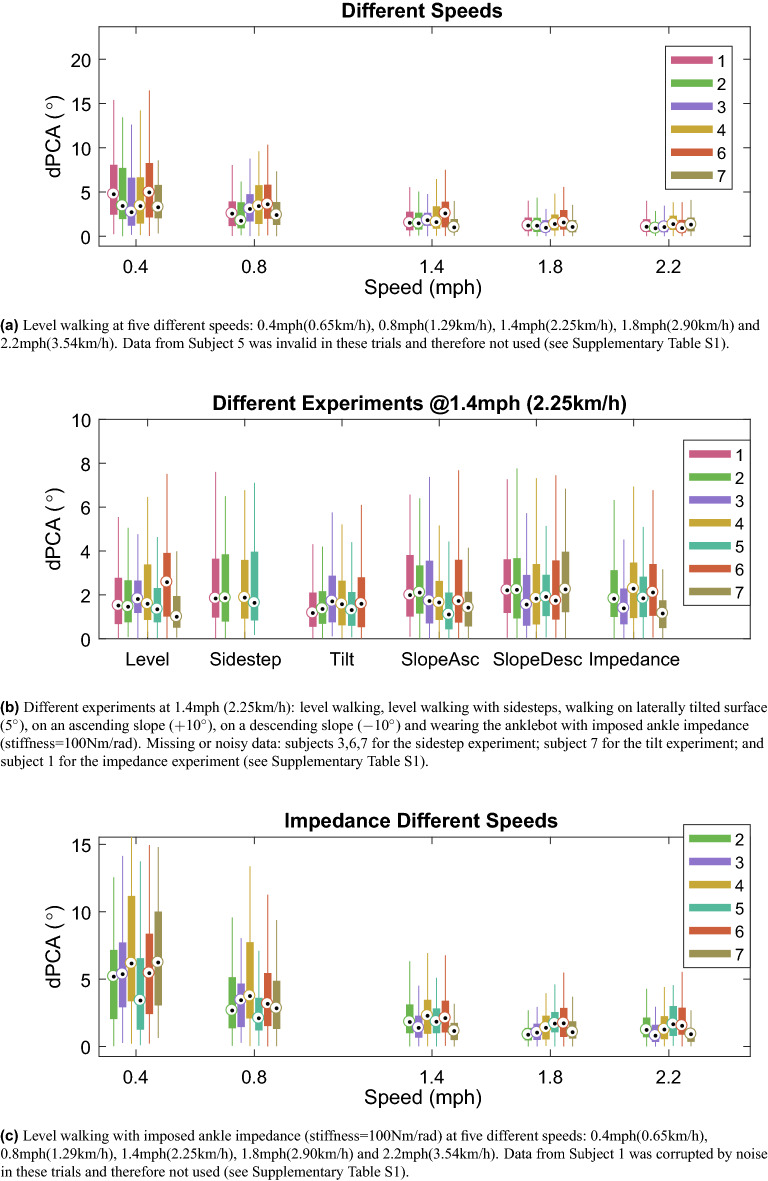
Box-plots of difference in angle between principal component of consecutive orbits (dPCA) for each subject.

The angular difference between the principal components of two consecutive orbits, dPCA, computed for the different experiments is represented as box-plots in Fig. 4. The median dPCA for level walking at different speeds is low across all subjects (Fig. 4a). Nonetheless, it shows greater variation at lower speeds ($\le 0.8$ mph $\sim 1.29$ km/h) than higher. Similar behavior is exhibited with imposed ankle impedance (Fig. 4c). The two-way repeated measures ANOVA for dPCA revealed a statistically significant effect of Speed (F$_{4,16}=139.830$, $p<0.001$, partial $\eta ^2=0.972$), and of ankle Impedance (F$_{1,4}=18.004$, $p=0.013$, partial $\eta ^2=0.819$). Furthermore, the interaction effect between Speed and ankle Impedance was significant (F$_{4,16}=9.075$, $p<0.001$, partial $\eta ^2=0.694$). The post-hoc pairwise comparison reveals that dPCA varied significantly with imposed ankle stiffness only at 0.4 mph (0.65 km/h), with mean difference of $2.03^\circ$. There is no significant difference between speeds 1.4, 1.8 and 2.2 mph (2.25, 2.90, 3.54 km/h) ($p > 0.10$), with or without imposed ankle stiffness, with dPCA around $1.32^\circ$. There is a significant difference between speed 0.4 mph (0.65 km/h) and 1.4, 1.8 and 2.2 mph (2.25, 2.90, 3.54 km/h), with or without imposed stiffness, with a mean difference of $2.15^\circ$ when no impedance is imposed, and of $4.12^\circ$ when ankle stiffness is imposed. For the case imposed impedance, there is a significant difference between speed 0.8 mph and all others (mean difference of $1.87^\circ$), whereas no significant difference is found between 0.8 mph (1.29 km/h) and any other speed when no ankle stiffness is added ($p > 0.06$, mean difference of $1.25^\circ$). The experiments performed at 1.4 mph (2.25 km/h) show little variation in dPCA across experiments and subjects (Fig. 4b). The ANOVA revealed no significant difference between experiment types (F$_{4, 16}$ = 1.530, $p=0.241$).

**Figure 5:**
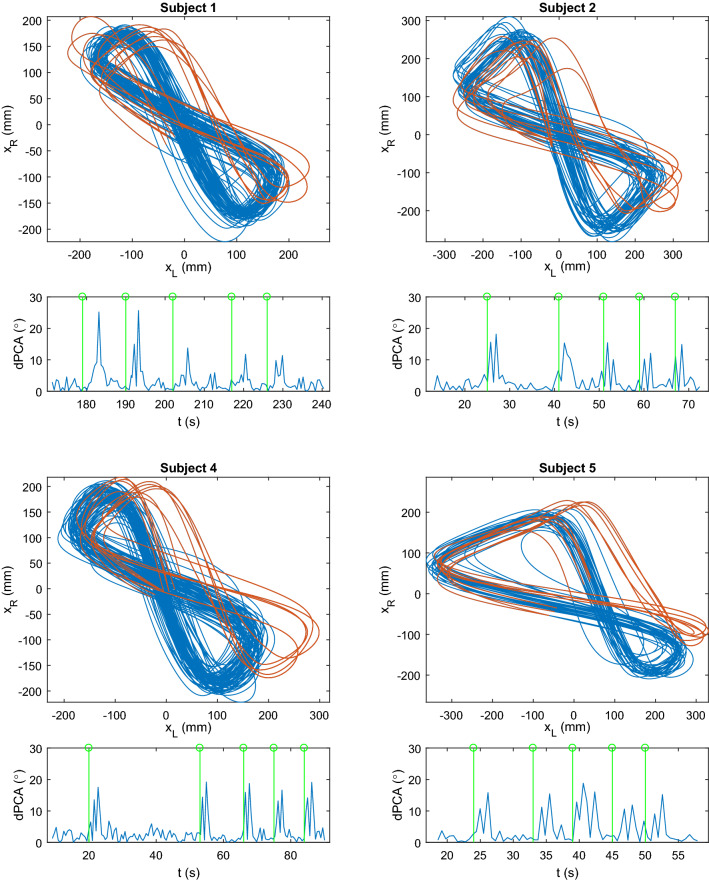
Feet spatial trajectory of four subjects walking on level-ground, interleaved with sidesteps. The angle between the principal components of the consecutive trajectories, dPCA, below the feet spatial trajectory, with the green lines corresponding to the time the sound signal is given to command the sidestep. The high dPCA values correspond to the sidestep discrete event (orange orbits), which disrupts the normal walking pattern (blue orbits).

For the sidestep experiment, the 2D spatial trajectory and dPCA for consecutive steps are shown in Fig. 5 for each subject. The green stems represent the time instants when an auditory cue was given to perform a sidestep. Sidesteps correspond to orbits that fall outside the rhythmic trajectory, resulting in an increased dPCA. Supplementary Fig. S4 shows an example of the time evolution of the forward position of both feet during a sidestep event.

### Validation of MSJR on simulated gait at different speeds

As in^34^, we show a simulated approach to the smoothness of a signal, now adapted for walking gait on a treadmill. Three consecutive steps (left-right-left) with constant amplitude $A_{step}$ and duration $d_{step}$ are taken at equally spaced time intervals and the MSJR is computed. Supplementary Fig. S5 shows representative simulated examples of walking gait at three different speeds.

At low speed there are clear periods of no movement, where the velocity is zero. As a result, the MSJ of this movement is much greater than that described by the purely sinusoidal movement, hence the MSJR is high. As speed increases, steps occur closer together, and the MSJR decreases. The middle plot shows the theoretical limit for which walking gait is a sequence of back-to-back discrete movements, where the acceleration still goes to zero at the transition between steps. Finally, at a higher speed, the beginning and end of consecutive steps start to overlap in time, resulting in a more rhythmic pattern, accurately represented by a lower MSJR.

**Figure 6:**
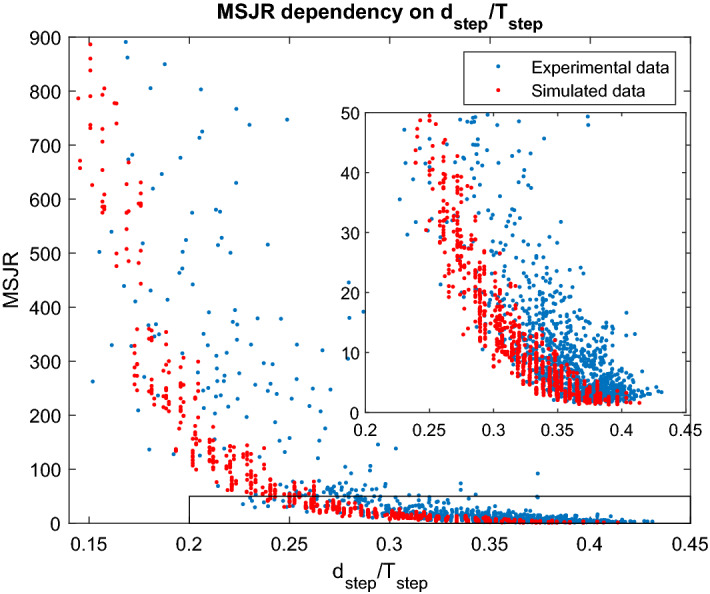
MSJR vs stride Swing/Period ratio from simulated and measured data; Blue: simulated gait for different speeds, with different step amplitudes and swing durations; Red: MSJR computed on data from subjects across five different speeds, fit to an exponential curve. Both simulated and measured MSJR exhibit an exponential decrease in MSJR with increased swing/period ratio, although the experimental data has a greater dispersion. Inner plot is zoomed to MSJR = (0, 50).

In the previous simulation conditions, we considered the three steps to occur at regular intervals, with the same amplitude and duration. Such approach leads to a symmetric profile when the relative position between the two feet is computed, for which a sine wave is a good fit. However, in actual gait such symmetry is hardly met, as observed in Fig. 2: steps will have different amplitudes and durations at a constant speed, which results in slight asymmetries in the position difference signal, including some periods of zero-velocity, and hence increasing the MSJR. In order to simulate different walking approaches to a given speed and determine the influence of $A_{step}$ and $d_{step}$ on MSJR, we follow the same procedure to generate walking gait, now adding uniform noise to the step duration (between [− 0.1, 0.1] s) and to the step amplitude (between [− 0.025, 0.025] m) on each foot individually and for each step. The resulting MSJR for the perturbed step amplitude and duration at different speed conditions is represented in Fig. 6, as a function of the ratio between step duration and stride period. These results show that the simulated MSJR decreases exponentially with the stride swing/period ratio. The exponential trend is in accordance with the measured data for different speeds across subjects, as shown in Fig. 1a, although with greater dispersion.

### Validation of dPCA on simulated gait with a sidestep

**Figure 7:**
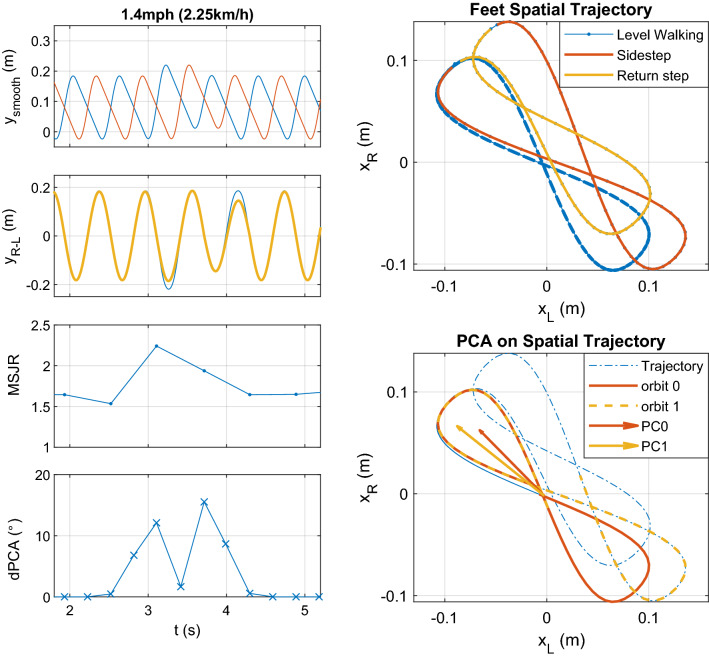
Simulated feet position evolution in time during treadmill level walking at constant speed $v_{treadmill}$ = 1.4 mph =2.25 km/h, amplitude $A_{step}$ = 0.37 m, step duration $d_{step}$ = 0.325 s, interleaved with a sidestep at $t=3$ s. The sidestep is represented by a step of increased amplitude $A_{sidestep}=1.1A_{step}$ first on the left foot, L (blue, top-left), then on the right, R (red, top-left). To resume normal walking, a step of amplitude 0.9$A_{step}$ is taken by the left then the right foot, which resumes the normal walking profile. $f_{\sin }$ (yellow, left, second row) approximates $y_{R-L}$ (blue, left, second row). MSJR and dPCA are presented for each step; Top-right plot shows the spatial trajectory of the two feet in level walking (blue), during the sidestep (orange) and the returning step (yellow); The difference between two orbits can be described by the angle between the principal component of the two, as shown in the bottom-right plot.

Based on the previous results (see Supplementary Fig. S5), the simulated speed of 1.4 mph (2.25 km/h) produces a rhythmic pattern. Therefore, a sequence of left and right steps is taken at constant speed, amplitude, $A_{step}$, and duration, $d_{step}$, at equally spaced time intervals. The following procedure is used to emulate a sidestep amidst this normal gait: at a given left foot step *k*, its amplitude is increased to $A_{step}^k=1.1A_{step}$ to simulate a sidestep; the following right foot step also has amplitude $A_{step}^k$, simulating a tandem step; then, the following left foot step $k+1$ has amplitude $A_{step}^{k+1}=0.9A_{step}$, followed by a right foot step of the same length, returning to normal gait. The simulated gait is presented in Fig. 7, showing both the forward position in time and the spatial trajectory of the feet. The MSJR and dPCA computed for the different strides in the simulated trial are also displayed. In the simulated trajectory in Fig. 7, the principal components of two consecutive orbits form an angle, which indicates the sidestep is present. The angle dPCA between the principal component of two consecutive orbits of this simulated trajectory is depicted in Fig. 7, bottom-left plot, which shows an increased angle on the onset of the sidestep and when returning to normal gait. The MSJR values for these strides are very low, even when the sidestep occurs.

## Discussion

The MSJR applied to the relative position between the two feet is able to capture the rhythmicity and discreteness of the walking gait in the several different walking conditions studied in this work. Considering level walking at different speeds (Fig. 1a), there is a common trend for each subject: MSJR is high for the slowest speed and becomes lower as the speed increases. At 0.4 mph (0.65 km/h), the high MSJR ($>100$), significantly different from higher speeds, shows that walking at such low speeds becomes a sequence of discrete movements. On the other hand, the low MSJR exhibited at $\ge 1.4$ mph (2.25 km/h) ($<20$), with no significant difference found between these speeds, but significantly different from lower speeds, indicates that gait at these speeds exhibits a more rhythmic behavior. The 0.8 mph (1.29 km/h) walking speed, significantly different from all other speeds, reveals itself as an interesting turning point for rhythmic and discrete movements. While some subjects exhibit MSJR $\in \left[ 10,40\right]$, within the range of values found at 1.4 mph (2.25 km/h), others present higher MSJR $\in \left[ 40,100\right]$. The simulated walking model is useful to better understand how speed influences the MSJR values. Simulated walking gait at different speeds with constant step amplitude and swing duration, represented in Supplementary Fig. S5, shows that as speed increases and the steps occur closer together, the MSJR decreases. The decrease in MSJR with speed is a consequence of an increase in the ratio between step duration and stride period: as the step duration approaches half of the period, the relative position between the two feet becomes more sinusoidal, hence the MSJR decreases. This relationship is observed when the MSJR is plotted against the stride swing/period ratio ($d_{step}/T_{step}$) for the experimental data (Fig. 6). Perturbations in the step amplitude and duration in the simulated model introduce asymmetries and lead to variations in MSJR for the same $d_{step}/T_{step}$.

The simulated model considers a smoothest discrete step and a final foot velocity that matches that of the treadmill. In practice, the step movement and foot contact with the ground will introduce a greater jerk, and hence greater MSRJ, than accounted for in the simulation. Nonetheless, for the experimental data in level walking at different speeds, $d_{step}/T_{step}$ in Fig. 3a and MSJR in Fig. 1a are consistent with the simulated model; lower speeds lead to smaller $d_{step}/T_{step}$ and higher MSJR, higher speeds result in increasing $d_{step}/T_{step}$ and lower MSJR. $d_{step}/T_{step}$ increases towards 0.5 for all subjects as the speed goes over 1.4 mph (2.25 km/h), remaining fairly constant for the three highest speeds. At this point, a step takes almost half of the stride period and the only way to increase the speed is increasing the step amplitude, which is consistent with the results in Fig. 3. At 0.8 mph (1.29 km/h), the subjects can adopt different strategies to maintain the pace, as shown by the plots of $d_{step}/T_{step}$ and amplitude (Fig. 3): either take larger steps but at a reduced frequency, resulting in lower $d_{step}/T_{step}$ ratio, which implied that the stance duration in a complete stride increases, or alternatively lower amplitude and more frequent steps (higher $d_{step}/T_{step}$ ratio). Subjects that adopt a higher $d_{step}/T_{step}$ (Subjects 1, 2, 4) have lower MSJR and also exhibit a smaller step amplitude than the other subjects. These results are coherent with the simulated gaits with different amplitudes and swing duration, where the MSJR decreases as $d_{step}/T_{step}$ increases (Fig. 6). Such results show that for the lowest speed, all subjects adopt a sequence of discrete steps strategy and that for speeds $\ge 1.4$ mph (2.25 km/h), gait is forcefully rhythmic. At 0.8 mph (1.29 km/h), gait for some subjects will remain a sequence of discrete steps, while others will transition to a more rhythmic movement. Harmonicity is consistently low for all subjects at slow speeds, as expected for discrete movements (Supplementary Fig. S2). However, as the speed increases, harmonicity median value is low and its variability high, ranging the entire spectrum of possible values, between 0 and 1, thus it is not a robust metric to assess the rhythmicity/discreteness of the walking gait. Rhythmicity is maintained for the different experiments at 1.4 mph (2.25 km/h) (Fig. 1b): low MSJR ($< 30$), consistent with the values in level walking at 1.4 mph (2.25 km/h), with no significant difference between experiments. Sidesteps do not introduce an increase in MSJR, which is supported by the simulation results (Fig. 7). As previously reported in^45^, when a discrete movement is introduced amidst a rhythmic behavior, it tends to occur at a preferred phase, synchronized with the ongoing rhythmic movement. In this case, the sidestep does not start in the ongoing swing phase. Rather, it is synchronized with the onset of a step, not changing the jerk profile significantly. Interestingly enough, while slopes and the laterally tilted surface introduce different joint coordination patterns, they achieve a similar foot position profile and thus MSJR is not altered by such perturbations. Finally, an externally imposed ankle stiffness (Fig. 1c) results in a similar gait pattern and MSJR values as observed in free level-ground walking across the different speeds (Fig. 1a), with no statistically significant difference due to imposed stiffness. Such results are coherent with the paradigm of dynamic primitives, where the mechanical impedance is added to a virtual trajectory, composed of rhythmic and discrete movements defined by the CNS, and so the imposed ankle impedance does not significantly change the planned virtual trajectory and hence rhythmicity.

The dPCA computed on the 2D spatial trajectory of both feet is able to identify discrete events that occur amidst rhythmic movements. Regarding level walking at different speeds, the median dPCA is low across all subjects (Fig. 4a). Similar behavior is exhibited with imposed ankle impedance (Fig. 4c), with significant difference found only for 0.4 mph due to imposed stiffness, with low mean difference ($2.03^\circ$). Nonetheless, it shows greater variation at lower speeds ($\le 0.8$ mph–1.29 km/h), revealing statistically significant difference between 0.4 mph and higher speeds, with or without imposed stiffness, and between 0.8 mph and all other speeds when ankle stiffness is imposed. Such result points to the greater difficulty in maintaining a steady, repeatable gait pattern at lower speeds, as seen in the 2D spatial trajectory corresponding to such speeds (Supplementary Fig. S3). The different experiments performed at 1.4 mph (2.25 km/h) also exhibit little variation in dPCA (no significant difference found between experiments), even for the tilt and slopes experiments (Fig. 4b), which shows that these different kinds of gait at this speed are highly repeatable. For the sidestep experiment, dPCA values are low throughout the trial except when a sidestep occurs, as seen in Fig. 5, suddenly increasing to values above $10^\circ$. Such events can result in an increase in step amplitude, swing speed or changes in timing of the step onset. These results are supported by simulation (Fig. 7), in which a sidestep results in an orbit outside the rhythmic pattern generated and in an increased dPCA.

This work introduces two new biomarkers to assess the level of discreteness or rhythmicity of movement in locomotion, filling a gap existing in the analysis of the purported dynamic primitives of movement in locomotion. A limitation of this study is the relatively small number of subjects included. Nevertheless, these biomarkers appear to accurately assess rhythmic and discrete movements in different walking conditions. The MSJR determines whether the movements are more rhythmic or discrete. In case rhythmicity is high, the dPCA allows to detect discrete movements occurring amidst the rhythmic movements, uncaptured by the MSJR, as they occur in phase with the onset of the step and do not change the jerk profile. These biomarkers should be evaluated on neurologically impaired subjects with gait disorders, as they can be helpful in understanding the extent and location of the lesion, but also in the design and assessment of the outcomes of therapy.

## Methods

### Experimental design

The focus was to illuminate how the primitives are combined. A reasonable assumption is that rhythmic movements are prominent in forward fast walking on flat ground and that as we slow down, eventually we transition to discrete movements. A single step to the side, e.g., to avoid an obstacle, introduces a discrete movement to be coupled with the ongoing rhythmic stepping. Controlling foot contact with the ground is enabled via the control of ankle impedance. Joint kinematics will be captured in the following conditions: (1) walking overground at different speeds, (2) walking with imposed joint impedance, (3) walking on ascending and descending slopes, (4) walking on a laterally tilted surface, (5) walking with side-stepping. The experiments were conducted on the MIT-Skywalker, a device which includes a novel body-weight support system^46^, duo split dynamic treadmill belts that reposition themselves in order to allow foot clearance, and a frontal rotator. The treadmill system contains five active degrees of freedom. Training can encompass a single primitive or all in order to meet the needs of each individual patient. For a more detailed description of the MIT Skywalker see^16^.

Selected experiments to this novel experiment are described:

1. Unperturbed Walking at Different Self-Selected Speeds: Subjects walked on the MIT-Skywalker at 5 different speeds: Comfortable speed of 1.4 mph ($\sim 2.25$ km/h), faster speeds of 1.8 mph ($\sim 2.90$ km/h) and 2.2 mph ($\sim 3.54$ km/h), slower speeds of 0.8 mph ($\sim 1.29$ km/h) and 0.4 mph ($\sim$ 0.65 km/h). Comfortable speed was selected based on a preliminary study on chronic neurological patients training on the MITSkywalker, in which they achieved an average self-selected speed of 1.4 mph ($\sim 2.25$ km/h) at train completion^16^. Subjects walked bare-footed. While this experiment aimed to elicit rhythmic movements, the slowing of walking speed was expected to induce a transition from smooth oscillatory rhythmic movements to discrete steps, i.e. submovements, as previously documented in arm movements^18,20^. To evaluate rhythmicity, we examined the time profiles of the feet. It was expected that at slower walking speeds there would be a transition from rhythmic to discrete movements.
2. Walking with Imposed Impedance: The interaction of oscillations with impedance was tested by wearing the anklebot^47^. The anklebot can be programmed to increase or decrease the impedance at the ankle. We programmed it to add a constant high stiffness of 100Nm/rad, a value greater than ankle stiffness during the swing phase, but lower than during the stance phase, and in particular, toe-off^19^. Subjects walked at the same 5 instructed speeds but wore the anklebot while walking. We examined whether changing the ankle stiffness affected walking speed and, in particular, the speed at which transitions between rhythmic and discrete movements occurred.
3. Walking on Ascending and Descending Slopes: Walking on non-horizontal surfaces imposes increased stress on the ankle joint and is expected to increase demands on impedance at the ankle, knee, and hip. In addition, by requiring slanted placement of the foot with respect to the tilted surface, an additional discrete element may be coupled to the oscillatory pattern of walking. Subjects walked on a treadmill that was raised to $10^\circ$ upward or $10^\circ$ downward slopes. They walked at a comfortable speed of 1.4 mph ($\sim 2.25$ km/h) for 2 trials for each condition. Slopes of were presented in random order. Analyses focused on changes at foot as a function of slope angle. In particular, the foot trajectories were examined for discrete elements induced by placing the foot.
4. Walking on Laterally Tilted Slopes: One scenario that requires explicit adaptation of ankle impedance is walking on laterally tilted slopes on the MIT-Skywalker. Subjects walked at a comfortable speed of 1.4 mph ($\sim 2.25$ km/h) on lateral slopes of $5^\circ$ lateral tilt configuration, again bare-footed. As the surface angle induced supination in one ankle and pronation in the other ankle, subjects walked in both tilts so that each foot experiences both deflections. Subjects performed 2 trials per condition. Changes in oscillation patterns due to changes of impedance as a function of inclination angle will be investigated.
5. Perturbations due to Side-Stepping: Subjects walked at a comfortable speed of 1.4 mph ($\sim 2.25$ km/h). An auditory signal instructed them to step sideways, as if avoiding an obstacle, i.e., they stepped with the left foot in front of the right (or vice-versa), perform a tandem step and then resume normal waking. The signal was given 5 times at random phases of the stepping cycle to study how such a discrete movement was integrated into steady-state rhythmic walking. Analyses assessed: (1) how the lateral step initiation depended on the signal’s phase, (2) whether the side-step affected the ongoing walking rhythm.

**Figure 8:**
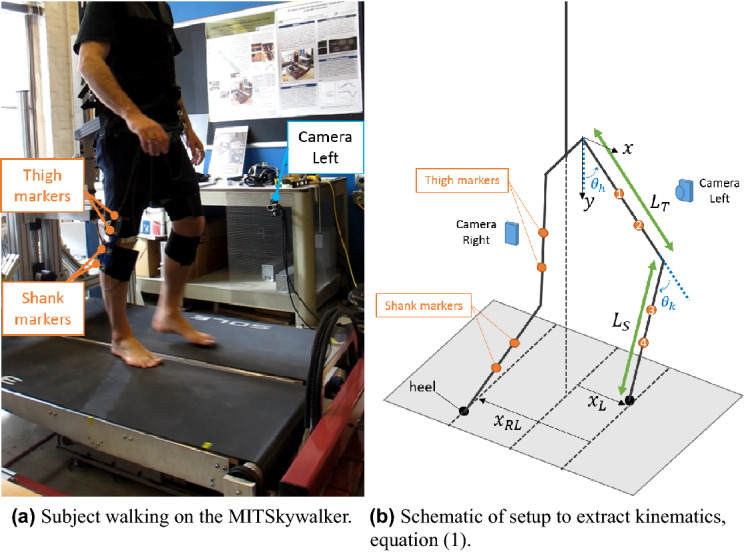
Experimental setup for kinematic data acquisition in walking gait on the MIT-Skywalker^16^.

All subjects walked, for about 1 min per trial, on the MIT Skywalker with bodyweight support added for safety (but not unloading), as shown in Fig. 8a.

### Experimental subjects

We enrolled seven healthy subjects (age: $32\pm 13$), following the suggestion of Poulton for the number of subjects^48^. The Massachusetts Institute of Technology (MIT) Committee on the Use of Humans as Experimental Subjects (COUHES) approved the study, in accordance with the Declaration of Helsinki. All volunteers gave written informed consent to participate.

### Data collection

Sagittal plane kinematic data was collected using two cameras located on either side of the treadmill, recording at 100 Hz. These cameras were used to track four infrared markers on either leg, two placed on the thigh (markers 1 and 2) and two on the shank (markers 3 and 4), as shown in Fig. 8b. The foot heel forward position in the sagittal plane, *x*, is then obtained from the hip and knee angles, $\theta _h$ and $\theta _k$, respectively, thigh and shank lengths, $L_T$ and $L_S$, and markers positions,$(x_i,y_i), i=\{1,2,3,4\}$, as described in Eq. (1)^16^.

1 $$\begin{aligned} \begin{aligned} \theta _h&= \arctan ((x_2-x_1)/(y_2-y_1))\\ \theta _k&= \theta _h -\arctan ((x_4-x_3)/(y_4-y_3))\\ x&= L_T\sin (\theta _h)+L_S \sin (\theta _h-\theta _k) \end{aligned} \end{aligned}$$

Notice that the computed forward position is with respect to the body’s midline, i.e., to the hip position, as shown in Fig. 8b.

The kinematic data was then processed in MATLAB (Mathworks, Natick, MA, USA). The position and angle data were filtered to remove outliers and then fitted to a quintic smoothing spline (*spaps()* function in MATLAB). The position derivatives (velocity, acceleration and jerk) were then computed from the spline and evaluated at the same time points as the position data. The complete acquired data is segmented such that each segment corresponds to a period of constant treadmill speed. For each data segment, the mean position is removed from both left and right foot position.

### Mean-squared Jerk Ratio (MSJR)

The smoothness of a rhythmic movement can be assessed by computing its mean-squared jerk (MSJ) and dividing it by that of a maximally smooth rhythmic movement, resulting in the unitless mean-squared jerk ratio (MSJR)^34^. The smoothness is computed according to Algorithm 1. First, we take the relative position between right and left foot along the trial, $x_{RL}(t)=x_R(t)-x_L(t)$, and remove the mean. Then, consecutive zero-crossings in $x_{RL}(t)$ with the same slope sign are used to segment $x_{RL}$ into movements, $x_{s_i}(t)$. Then, the MSJ of each movement, $\text {msj}_{x_{s_i}}$, is computed. The amplitude, $A_{\sin }$, and the period, $T_{\sin }$, of the maximally smooth rhythmic movement, a sine function, $f_{\sin _i}$, is extracted from $x_{s_i}(t)$, as done in^20^: $T_{\sin }$ matches the duration of $x_{s_i}$, $A_{\sin }$ is the minimum value between the absolute values of the minimum and maximum of $x_{s_i}(t)$, $A_{\sin } = \min (\Vert \max (x_{s_i})\Vert, \Vert \min (x_{s_i})\Vert )$. The mean-squared jerk of a sine function over one period is $\text {msj}_{f_{\sin } }=0.5A_{\sin }^2 \omega _{\sin }^6,\omega _{\sin }=2\pi /T_{\sin }$. The mean-squared jerk ratio $\text {MSJR}= \text {msj}_{x_{s_i } }/\text {msj}_{f_{\sin }} \ge 1$ will be high for a movement far from a smooth rhythmic movement, drawing closer to 1 as the movement becomes smoother.
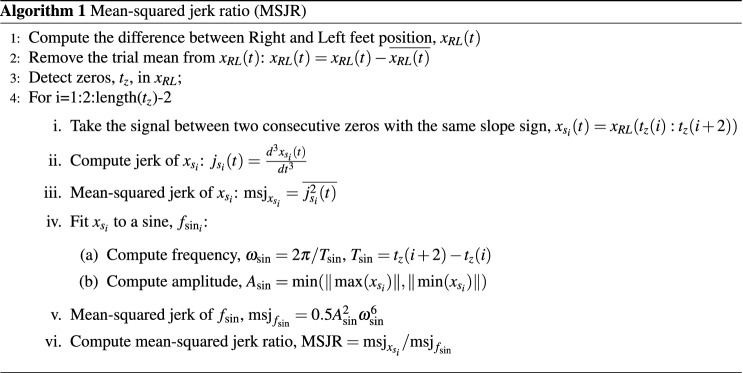


Guiard^33^ defined the concept of harmonicity as a measure of rhythmicity or discreteness for movements of the (single) upper limb. Harmonicity is computed on $x_{s_i}(t)$ and compared with the MSJR. Consider a window defined by two consecutive zero-crossings in displacement and extract the maximum and minimum acceleration peaks in the window. If there is only one peak, harmonicity is 1; if the maximum and minimum peaks have opposite signs, harmonicity is 0; otherwise, harmonicity is defined as the ratio between the absolute value of the minimum and the maximum acceleration peaks. As harmonicity approaches 0, the movement is considered to be predominantly discrete, whereas more rhythmic movements will have values closer to 1.

### Angular difference between principal components of two consecutive orbits (dPCA)

The plot of the right versus left foot forward position results in a figure-8-like spatial pattern. An orbit is defined between the points where the distance between the feet is zero ($x_{RL}=0$) and the slope of the relative position function $x_{RL} (t)$ has the same sign. The algorithm to compute the angle, dPCA, between the principal component of two consecutive orbits is described in Algorithm 2. As done to compute the MSJR, we take the relative position between right and left foot along the trial, $x_{RL} (t)$, and remove the mean. Then, consecutive zero-crossings in $x_{RL} (t)$ with the same slope sign are used to segment the right-left foot orbit $(x_{Rs}^i,x_{Ls}^i)$. A principal component analysis (PCA) is performed on this orbit to extract the first principal component vector, $v_{PCA}^i$. Finally, the angle dPCA between $v_{PCA}^i$ and the first component vector of the previous orbit, $v_{PCA}^{i-1}$ is computed.
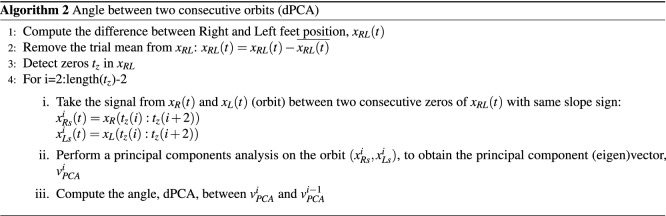


### Statistical analysis

Separate two-way repeated-measures analysis of variance (ANOVA) were conducted for MSJR and dPCA, with Speed (five speeds) and Ankle Impedance (with or without imposed ankle stiffness) as within-subjects factors. The interaction between speed and ankle impedance was examined. Additionally, separate one-way repeated-measures ANOVA were conducted for MSJR and dPCA, with Different Experiments (Level, Tilt, Slope Ascending, Slope Descending, Ankle Impedance) at 1.4 mph (2.25 km/h) as a within-subjects factor. The Greenhouse–Geisser correction factor was applied to the within subjects effects and a Bonferroni adjustment was applied to compensate for multiple comparisons. Significance was set at $\alpha =0.05$ for all statistical tests. The median of the dependent variables (MSJR and dPCA) over strides was used in the statistical analysis.

### Simulation of foot forward position in treadmill walking

Assuming a step amplitude $A_{step}$ and duration $d_{step}$, it is possible to trace the forward position, velocity and acceleration profiles of the foot walking on a treadmill at speed $v_{treadmill}$. For the shape of a step, let us assume a smoothest discrete movement $f_{sm}(t)$, starting at $t=t_0$, with amplitude $A_{step}$ and duration $d_{step}$^34^,

2 $$\begin{aligned} f_{sm}(t)&=A_{step} \left( 10\left( \frac{t-t_0}{d_{step}} \right) ^3 -15\left( \frac{t-t_0}{d_{step}} \right) ^4 +6\left( \frac{t-t_0}{d_{step}} \right) ^5 \right) \end{aligned}$$

The foot movement starts at $t=t_0$ with heel-off, followed by toe-off, initiating the swing phase, which ends with the heel-strike. Since the experiments are conducted on a treadmill, the simulated model considers that, during the stance phase, the foot is pulled back by the treadmill at constant speed, hence the initial velocity of the movement is equal to the treadmill speed, $-v_{treadmill}$. The model also considers that the final velocity upon heel-strike matches the treadmill speed, representing foot retraction that occurs naturally in walking. During foot-flat, $t-t_0 > d_{step}$, the foot movement is solely imposed by the treadmill movement. A complete stride, $y_{smooth}$, starting at $t=t_0$ at position $y_0$, is then described by,

3 $$\begin{aligned} y_{smooth}(t)&=\left\{ \begin{array}{ll} y_0-v_{treadmill} t +f_{sm}(t), &{}\quad 0 \le t-t_0\le d_{step} \\ y_0-v_{treadmill} t +A_{step}, &{}\quad t-t_0 > d_{step} \end{array}\right. \end{aligned}$$

Such simplified model can be used to better understand how the metrics developed are affected by the changes in parameters, namely the step amplitude, swing duration and speed.

## Supplementary Information

Supplementary Information.

## Data Availability

We anticipate that the data captured and created by this research will be of broad interest to communities engaged in research on human motor behavior. Data generated by this research project will be made publicly accessible through a portal linked to our lab homepage http://the77lab.mit.edu/. Access to these data will be “read-only” and password protected. Passwords will be made freely available upon submission of a request by email agreement that the source of the data will be acknowledged in any publication arising from use of these data. Please contact H. I. Krebs to request data access.

## References

[1] George MG (2017). CDC Grand Rounds: Public health strategies to prevent and treat strokes. MMWR Morb. Mortal. Wkly. Rep..

[2] Good DC (1994). Treatment strategies for enhancing motor recovery in stroke rehabilitation. Neurorehabilit. Neural Repair.

[3] Kleim JA, Jones TA (2008). Principles of experience-dependent neural plasticity: Implications for rehabilitation after brain damage. J. Speech Lang. Hear. Res..

[4] Winstein CJ (2016). Guidelines for adult stroke rehabilitation and recovery: A guideline for healthcare professionals from the American Heart Association/American Stroke Association. Stroke.

[5] Miller EL (2010). Comprehensive overview of nursing and interdisciplinary rehabilitation care of the stroke patient: A scientific statement from the American Heart Association. Stroke.

[6] Hogan, N., Krebs, H., Charnnarong, J., Srikrishna, P. & Sharon, A. MIT-MANUS: A workstation for manual therapy and training. I. In *Proceedings IEEE International Workshop on Robot and Human Communication*, 161–165. 10.1109/ROMAN.1992.253895 (IEEE, 1992).

[7] Krebs H, Hogan N, Aisen M, Volpe B (1998). Robot-aided neurorehabilitation. IEEE Trans. Rehabil. Eng..

[8] Krebs HI (2007). Robot-aided neurorehabilitation: A robot for wrist rehabilitation. IEEE Trans. Neural Syst. Rehabil. Eng..

[9] Aisen ML, Krebs HI, Hogan N, McDowell F, Volpe BT (1997). The effect of robot-assisted therapy and rehabilitative training on motor recovery following stroke. Arch. Neurol..

[10] Volpe B (1999). Robot training enhanced motor outcome in patients with stroke maintained over 3 years. Neurology.

[11] Ferraro M (2002). Assessing the motor status score: A scale for the evaluation of upper limb motor outcomes in patients after stroke. Neurorehabilit. Neural Repair.

[12] Lo AC (2010). Robot-assisted therapy for long-term upper-limb impairment after stroke. N. Engl. J. Med..

[13] Wu X, Guarino P, Lo AC, Peduzzi P, Wininger M (2016). Long-term effectiveness of intensive therapy in chronic stroke. Neurorehabilit. Neural Repair.

[14] Rodgers H (2019). Robot assisted training for the upper limb after stroke (RATULS): A multicentre randomised controlled trial. Lancet.

[15] Management of Stroke Rehabilitation Working Group (2010). VA/DOD clinical practice guideline for the management of stroke rehabilitation. J. Rehabil. Res. Dev..

[16] Susko T, Swaminathan K, Krebs HI (2016). MIT-Skywalker: A novel gait neurorehabilitation robot for stroke and cerebral palsy. IEEE Trans. Neural Syst. Rehabil. Eng..

[17] Hogan N, Sternad D (2013). Dynamic primitives in the control of locomotion. Front. Comput. Neurosci..

[18] Krebs HI, Aisen ML, Volpe BT, Hogan N (1999). Quantization of continuous arm movements in humans with brain injury. Proc. Natl. Acad. Sci. U.S.A..

[19] Lee H, Rouse EJ, Krebs HI (2016). Summary of human ankle mechanical impedance during walking. IEEE J. Transl. Eng. Health Med..

[20] Levy-Tzedek S, Krebs HI, Song D, Hogan N, Poizner H (2010). Non-monotonicity on a spatio-temporally defined cyclic task: Evidence of two movement types?. Exp. Brain Res..

[21] Hogan N, Sternad D (2012). Dynamic primitives of motor behavior. Biol. Cybern..

[22] Hogan N, Laumond J-P, Mansard N, Lasserre J-B (2017). Physical interaction via dynamic primitives. Geometric and Numerical Foundations of Movements.

[23] Lee H, Krebs HI, Hogan N (2014). Multivariable dynamic ankle mechanical impedance with relaxed muscles. IEEE Trans. Neural Syst. Rehabil. Eng..

[24] Lee H, Ho P, Rastgaar M, Krebs HI, Hogan N (2014). Multivariable static ankle mechanical impedance with active muscles. IEEE Trans. Neural Syst. Rehabil. Eng..

[25] Lee H, Hogan N (2015). Time-varying ankle mechanical impedance during human locomotion. IEEE Trans. Neural Syst. Rehabil. Eng..

[26] Moura Coelho R, Durand S, Martins J, Igo Krebs H (2022). Multivariable passive ankle impedance in stroke patients: A preliminary study. J. Biomech..

[27] Shorter AL (2021). Characterization and clinical implications of ankle impedance during walking in chronic stroke. Nat. Sci. Rep..

[28] Schaal S, Sternad D, Osu R, Kawato M (2004). Rhythmic arm movement is not discrete. Nat. Neurosci..

[29] Leconte P, OrbandeXivry JJ, Stoquart G, Lejeune T, Ronsse R (2016). Rhythmic arm movements are less affected than discrete ones after a stroke. Exp. Brain Res..

[30] Buchanan JJ, Park JH, Shea CH (2006). Target width scaling in a repetitive aiming task: Switching between cyclical and discrete units of action. Exp. Brain Res..

[31] van Mourik AM, Beek PJ (2004). Discrete and cyclical movements: Unified dynamics or separate control?. Acta Psychol..

[32] Smits-Engelsman B, Van Galen G, Duysens J (2002). The breakdown of Fitts’ law in rapid, reciprocal aiming movements. Exp. Brain Res..

[33] Guiard Y (1993). On Fitts’s and Hooke’s laws: Simple harmonic movement in upper-limb cyclical aiming. Acta Psychol..

[34] Hogan N, Sternad D (2007). On rhythmic and discrete movements: Reflections, definitions and implications for motor control. Exp. Brain Res..

[35] Perring S, Summers T (2007). Laboratory-free measurement of gait rhythmicity in the assessment of the degree of impairment and the effectiveness of rehabilitation in patients with vertigo resulting from vestibular hypofunction. Physiol. Meas..

[36] Frenkel-Toledo S (2005). Effect of gait speed on gait rhythmicity in Parkinson’s disease: Variability of stride time and swing time respond differently. J. NeuroEng. Rehabil..

[37] Plotnik M, Giladi N, Hausdorff JM (2007). A new measure for quantifying the bilateral coordination of human gait: Effects of aging and Parkinson’s disease. Exp. Brain Res..

[38] Yogev G (2005). Dual tasking, gait rhythmicity, and Parkinson’s disease: Which aspects of gait are attention demanding?. Eur. J. Neurosci..

[39] Baltadjieva R, Giladi N, Gruendlinger L, Peretz C, Hausdorff JM (2006). Marked alterations in the gait timing and rhythmicity of patients with de novo Parkinson’s disease. Eur. J. Neurosci..

[40] Hausdorff JM (2007). Rhythmic auditory stimulation modulates gait variability in Parkinson’s disease. Eur. J. Neurosci..

[41] Jackson, B. L., Coelho, R. M., Hirai, H. & Krebs, H. I. An investigation into rhythmic and discrete gait using the MIT Skywalker. In *2018 7th IEEE International Conference on Biomedical Robotics and Biomechatronics (Biorob)*, 922–927. 10.1109/BIOROB.2018.8488051 (2018).

[42] Ivanenko YP, Grasso R, Macellari V, Lacquaniti F (2002). Control of foot trajectory in human locomotion: Role of ground contact forces in simulated reduced gravity. J. Neurophysiol..

[43] Grasso R (2004). Distributed plasticity of locomotor pattern generators in spinal cord injured patients. Brain.

[44] Goswami A (1998). A new gait parameterization technique by means of cyclogram moments: Application to human slope walking. Gait Posture.

[45] de Rugy A, Sternad D (2003). Interaction between discrete and rhythmic movements: Reaction time and phase of discrete movement initiation during oscillatory movements. Brain Res..

[46] Gonçalves RS, Krebs HI (2017). MIT-Skywalker: Considerations on the design of a body weight support system. J. NeuroEng. Rehabil..

[47] Roy A (2009). Robot-aided neurorehabilitation: A novel robot for ankle rehabilitation. IEEE Trans. Robot..

[48] Poulton EC (1974). Tracking Skill and Manual Control.

