# Supplementary material for: Biomarkers for rhythmic and discrete dynamic primitives in locomotion
Source: Sci Rep. 2022 Nov 23;12:20165. doi: 10.1038/s41598-022-24565-z (PMC9691711; doi:10.1038/s41598-022-24565-z)
Supplement: Supplementary file 1 — Supplementary Information. [file 41598_2022_24565_MOESM1_ESM.pdf]

Supplementary Information

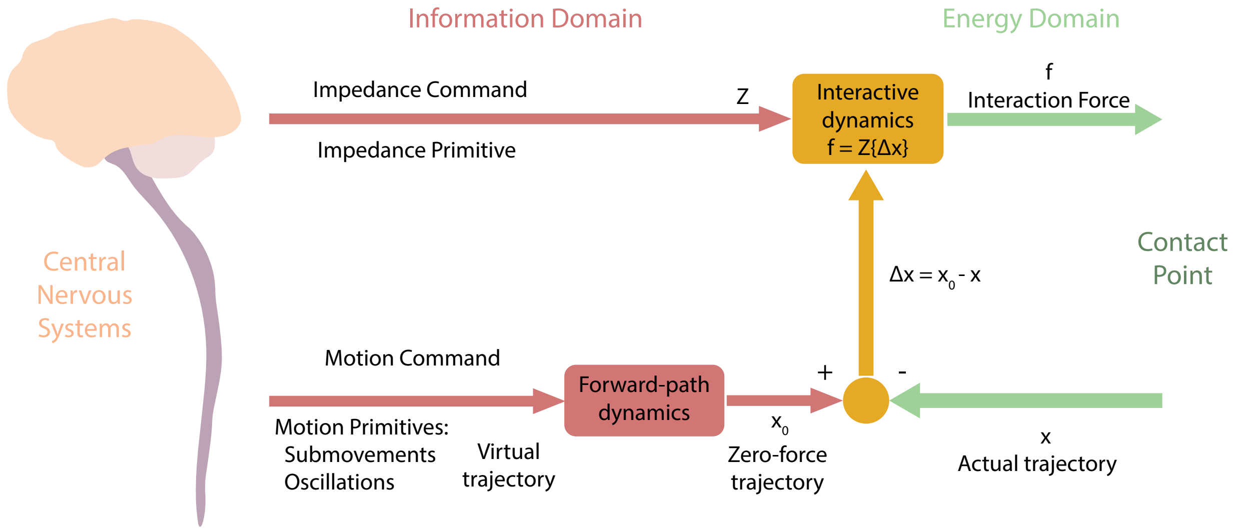

Supplementary Figure S1. Dynamic Primitives Model. To generate motion, we consider the conjecture that the CNS is commanding a virtual trajectory for the limbs to follow, composed of a combination of submovements and/or oscillations[18, 20]. A mechanical impedance primitive drives the limb towards the virtual trajectory and might also account for stable contact with the environment.

Experiment	Speed (mph / km/h)	Subject 1	2	3	4	5	6	7
Level	0.4 / 0.65	27	23	18	27	I	33	27
	0.8 / 1.29	46	25	38	31	I	43	60
	1.4 / 2.25	78	47	48	58	57	55	56
	1.8 / 2.90	72	56	81	54	I	47	61
	2.2 / 3.54	88	37	57	49	I	50	57
Sidestep	1.4 / 2.25	68	45	N	76	36	N	M
Tilt	1.4 / 2.25	59	45	47	58	57	54	M
Slope Ascent	1.4 / 2.25	48	26	57	87	59	91	47
Slope Descent	1.4 / 2.25	62	58	51	73	66	62	63
Imposed Impedance	0.4 / 0.65	N	23	19	23	32	24	15
	0.8 / 1.29	N	38	33	33	37	42	37
	1.4 / 2.25	N	44	46	58	50	57	60
	1.8 / 2.90	N	41	39	43	42	46	79
	2.2 / 3.54	N	43	41	51	44	37	46

Supplementary Table S1. Number of strides included in the analysis for each subject and experiment. Subject 7 did not perform the sidestep nor the tilt task (missing data, M). During the level walking experiment, the optical markers on Subject 5 slid, rendering the gathered data invalid (I). Three other experiments were excluded due to noise corruption (N).

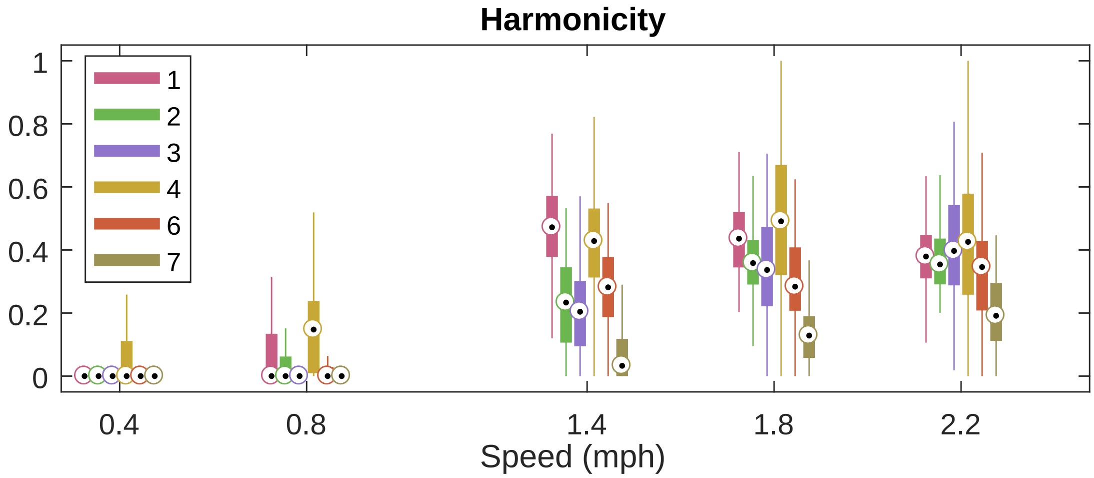

Supplementary Figure S2. Harmonicity computed on x_{RL} signal for each subject (data from Subject 5 was invalid, see Supplementary Table S1), for five speeds: 0.4mph(0.65km/h), 0.8mph(1.29km/h), 1.4mph(2.25km/h), 1.8mph(2.90km/h) and 2.2mph(3.54km/h). Although for the lower speeds, harmonicity is very low, which correctly describes the discreteness of the movements at those speeds, as speed increases, harmonicity has a low median value and high variability, almost spanning the complete range of possible values (between 0 and 1).

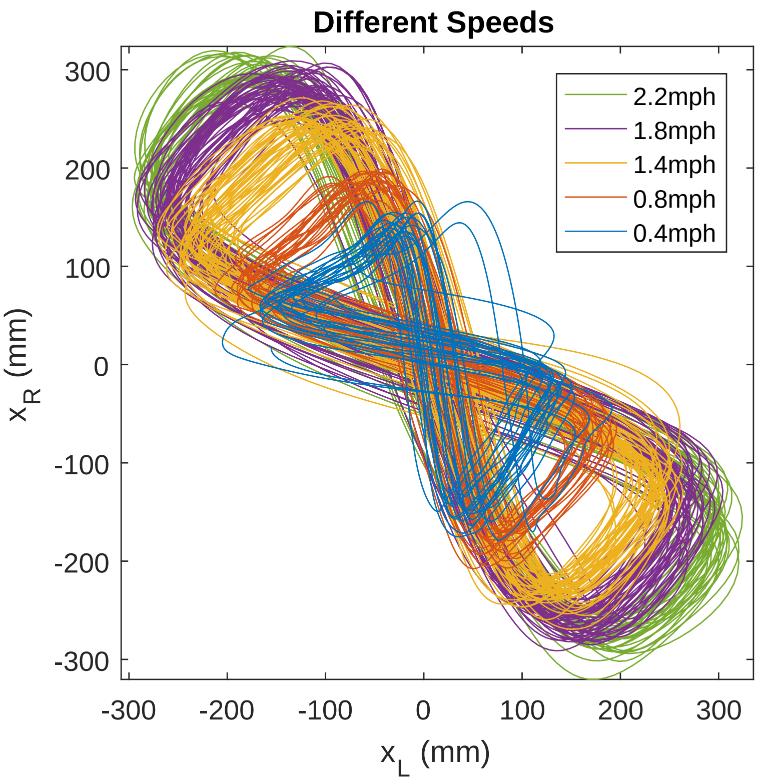

Supplementary Figure S3. 2D spatial trajectory generated by the forward position of the left (x_L) and right (x_R) foot of Subject 2 walking at different speeds: 0.4mph(0.65km/h), 0.8mph(1.29km/h), 1.4mph(2.25km/h), 1.8mph(2.90km/h) and 2.2mph(3.54km/h). A clear figure-eight-like pattern emerges, more consistent as the speed increases.

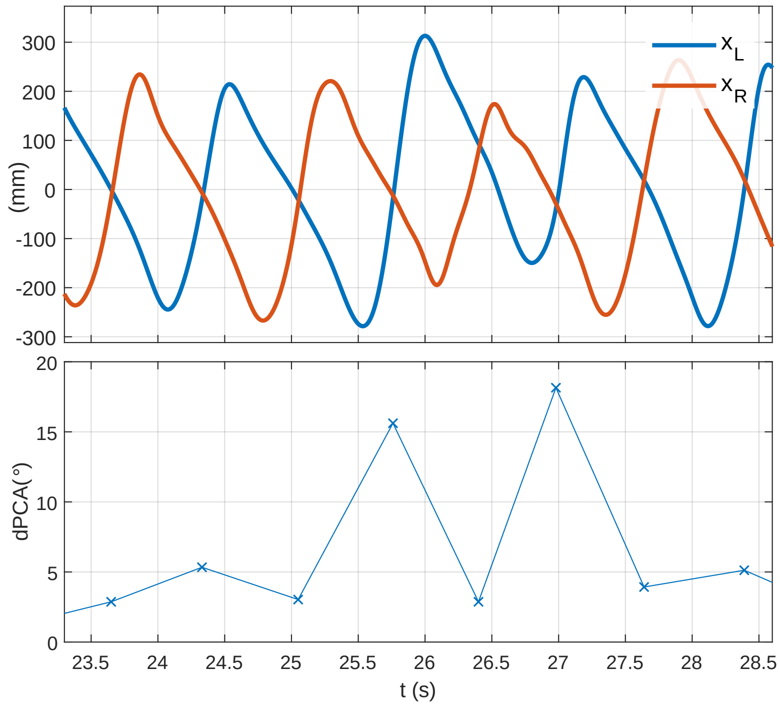

Supplementary Figure S4. Forward position of the left (x_L) and right (x_R) foot of Subject 2 walking at 1.4mph(2.25km/h), performing the sidestep experiment, and resulting dPCA. The sidestep and the return step deviate from the normal pattern, resulting in an increased dPCA.

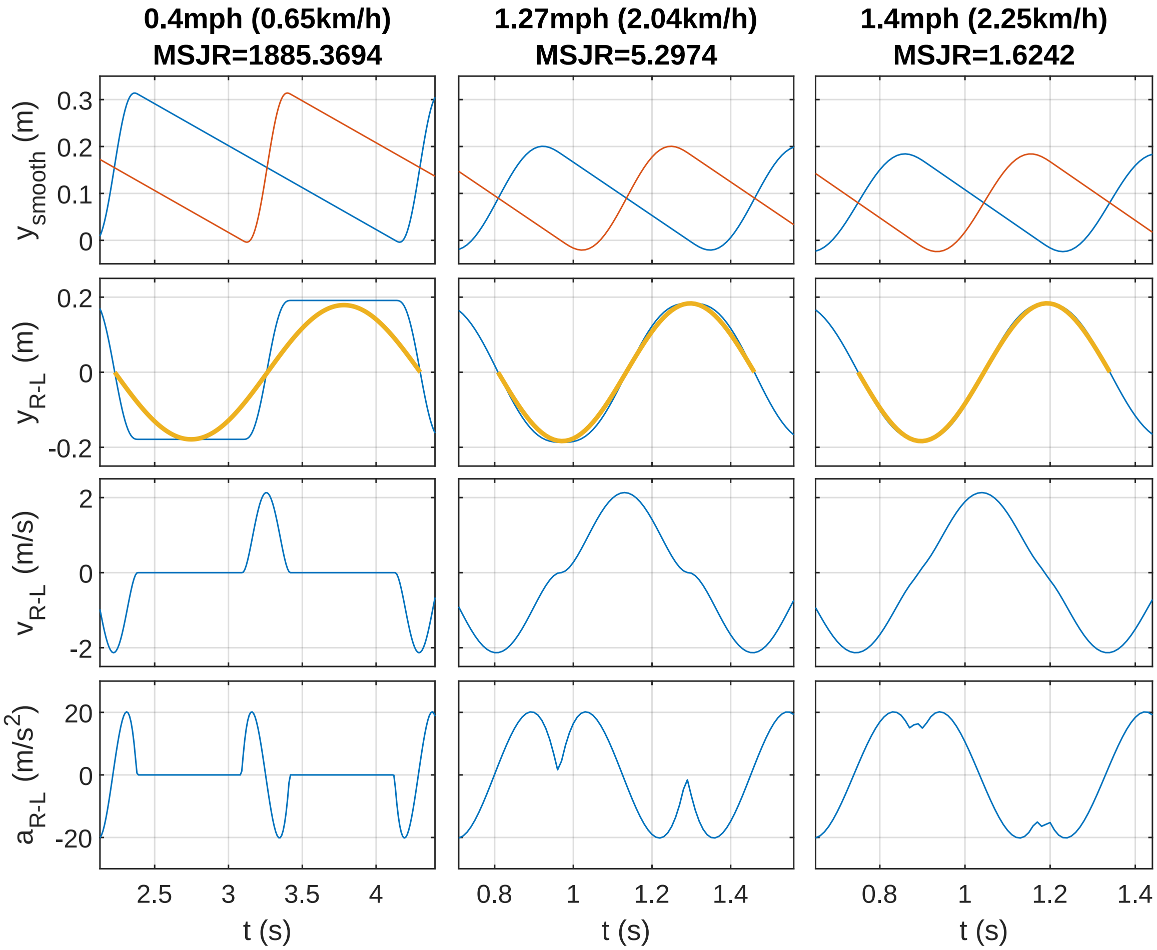

Supplementary Figure S5. Simulated walking gait steps at 3 different speeds, with constant step amplitude $A_{step} = 0.37$m and duration $d_{step} = 0.325$s. The top row shows the position of the Left (blue) and Right (red) foot. The second row shows the difference between Right and Left foot position, y_{R-L} (blue line). It is also shown the sine function that best fits y_{R-L}, $f_{\sin}$ (yellow).
